# SASD: the Synthetic Alternative Splicing Database for identifying novel isoform from proteomics

**DOI:** 10.1186/1471-2105-14-S14-S13

**Published:** 2013-10-09

**Authors:** Fan Zhang, Renee Drabier

**Affiliations:** 1 Department of Academic and Institutional Resources and Technology, University of North Texas Health Science Center, Fort Worth, USA; 2 Department of Forensic and Investigative Genetics, University of North Texas Health Science Center, Fort Worth, USA

## Abstract

**Background:**

Alternative splicing is an important and widespread mechanism for generating protein diversity and regulating protein expression. High-throughput identification and analysis of alternative splicing in the protein level has more advantages than in the mRNA level. The combination of alternative splicing database and tandem mass spectrometry provides a powerful technique for identification, analysis and characterization of potential novel alternative splicing protein isoforms from proteomics.

Therefore, based on the peptidomic database of human protein isoforms for proteomics experiments, our objective is to design a new alternative splicing database to 1) provide more coverage of genes, transcripts and alternative splicing, 2) exclusively focus on the alternative splicing, and 3) perform context-specific alternative splicing analysis.

**Results:**

We used a three-step pipeline to create a synthetic alternative splicing database (SASD) to identify novel alternative splicing isoforms and interpret them at the context of pathway, disease, drug and organ specificity or custom gene set with maximum coverage and exclusive focus on alternative splicing. First, we extracted information on gene structures of all genes in the Ensembl Genes 71 database and incorporated the Integrated Pathway Analysis Database. Then, we compiled artificial splicing transcripts. Lastly, we translated the artificial transcripts into alternative splicing peptides.

The SASD is a comprehensive database containing 56,630 genes (Ensembl gene IDs), 95,260 transcripts (Ensembl transcript IDs), and 11,919,779 Alternative Splicing peptides, and also covering about 1,956 pathways, 6,704 diseases, 5,615 drugs, and 52 organs. The database has a web-based user interface that allows users to search, display and download a single gene/transcript/protein, custom gene set, pathway, disease, drug, organ related alternative splicing. Moreover, the quality of the database was validated with comparison to other known databases and two case studies: 1) in liver cancer and 2) in breast cancer.

**Conclusions:**

The SASD provides the scientific community with an efficient means to identify, analyze, and characterize novel Exon Skipping and Intron Retention protein isoforms from mass spectrometry and interpret them at the context of pathway, disease, drug and organ specificity or custom gene set with maximum coverage and exclusive focus on alternative splicing.

## Background

Alternative splicing is a widespread mechanism for generating protein diversity and regulating protein expression with multiple splice isoforms. It was thought that at least 40-60% of human genes underwent alternative splicing to encode two or more splice isoforms [1]. Recent advances in high-throughput technologies have facilitated studies of genome-wide alternative splicing. These studies estimate that the prevalent post-transcriptional gene regulation mechanism affects greater than 95% of roughly 61,000 human genes and multiple regulatory processes, including chromatin modification and signal transduction [2]. Furthermore, there are evidences for alternatively splicing events that are often differentially regulated across tissue types and developmental stages, as well as among individuals and populations, suggesting that individual isoforms may serve specific spatial or temporal roles [3-5].

Alternative splicing is known to be involved in the regulation of normal physiological functions as well as pathologies. The alternative splicing isoform represents a new class of diagnostic biomarkers. Not only alternative splicing is thought to increase protein diversity of genomes, but also it has been found that splicing variants have been associated with numerous disease development and cancer cell growth. For example, David *et al. *found that aberrant expression of the splicing factors PTB, hnRNPA1 and hnRNPA2, regulated by the c-Myc oncogene, was responsible for the PKM1 to PKM2 switch in cancer [6]. This work helped us understand the alternative splicing's role in the cancer cell growth. Eswaran *et al. *systematically revealed splicing signatures of the three most common types of breast tumors using RNA sequencing: TNBC, non-TNBC and HER2-positive breast cancer and discovered subtype specific differentially spliced genes and splice isoforms not previously recognized in human transcriptome. They validated the presence of novel hybrid isoforms of critical molecules like CDK4, LARP1, ADD3, and PHLPP2 and found that exon skip and intron retention are predominant splice events in breast cancer [7]. Yae *et al. *found that epithelial splicing regulatory protein 1 regulates the expression of a CD44 variant isoform (CD44v), and knockdown of epithelial splicing regulatory protein 1 in CD44v+ cells results in an isoform switch from CD44v to CD44 standard (CD44s), leading to reduced cell surface expression of xCT and suppression of lung colonization. They suggested that the epithelial splicing regulatory protein 1-CD44v-xCT axis was thus a potential therapeutic target for the prevention of metastasis [8].

Recent methodological advances, including EST sequencing, exon array, exon-exon junction array, and next-generation sequencing of all mRNA transcripts, have made it possible to perform high-throughput alternative splicing analysis [7]. However, high-throughput identification and analysis of alternative splicing in the protein level has several advantages. For example, mRNA abundance in a cell often correlates poorly with the amount of protein synthesized, and proteins rather than mRNA transcripts are the actual major effector molecules in the cell.

The combination of alternative splicing database and tandem mass spectrometry provides a powerful technique for identification, analysis and characterization of potential novel alternative splicing protein isoforms from proteomics. In recent years, liquid chromatography tandem mass spectrometry (LC-MS/MS) has emerged as an innovative analytical technology applicable to a wide number of analyses including high-throughput identification of proteins [9]. LC-MS/MS proteomics has been used to identify candidate molecular biomarkers in diverse range of samples, including cells, tissues, serum/plasma, and other types of body fluids. Due to the inherent high variability of both clinical samples and MS/MS instruments, it is still challenging to quantify minute changes of proteins that exist in trace amount in response to changes in disease states of biological samples. Identifying alternative splicing isoform relevant to disease can improve both sensitivity and specificity of candidate disease biomarkers because many proteins could generate abundant alternative splicing isoforms in a disease, some of them may be exclusively regulated in a given disease condition, and therefore their identification process is often sufficient to distinguish between disease samples and controls [10].

However, without a proper alternative splicing database, tandem mass spectrometry could not discriminate against novel alternative splicing peptides [10,11]. Searching traditional protein sequence databases which are commonly used by peptide/protein search engine such as 1) IPI [12], 2) NCBI nr (ftp://ftp.ncbi.nih.gov/blast/db/FASTA/nr.gz), and 3) UniProt [13] biases the results towards well-understood protein isoforms because they contains a rather small set of splicing peptides and not enough for the identification of alternative splicing isoform from mass spectrometry data.

There are also currently several alternative splicing databases, for example, ASTD [14], EID [15,16], Fast DB [17], and ECgene [18]. They are not suitable for being directly applied to novel alternative splicing isoform identification without proper modifications made in format and content, because either their coverages are all relatively small in possible combination of alternative splicing junctions such as intron-exon, exon-intron, or non-neighboring exon, or single intron, or their storage formats make the databases difficult to use for mass spectrometry analysis and alternative splicing analysis.

Therefore, there is an urgent need to build an alternative splicing database which can be used by tandem mass spectrometry to identify the novel alternative splicing isoform. In 2010, we developed the PEPtidomics Protein Isoform Database (PEPPI [10], http://bio.informatics.iupui.edu/peppi), a database of computationally-synthesized human peptides that can identify protein isoforms derived from either alternatively spliced mRNA transcripts or SNP variations. We collected genome, pre-mRNA alternative splicing and SNP information from Ensembl and synthesized *in silico *isoform transcripts that cover all exons and theoretically possible junctions of exons and introns, as well as all their variations derived from known SNPs.

Based on the PEPPI [10], our objective is to design a new alternative splicing database to 1) provide more coverage of genes, transcripts and alternative splicing, 2) exclusively focus on the alternative splicing (we will build another database excusive to SNP isoform), and 3) perform context-specific alternative splicing analysis. More coverage means more sensitivity in identifying novel alternative splicing isoforms. Exclusive focus on alternative splicing can increase the specificity of the identification of alternative splicing. Context specificity analysis can improve our understanding of alternative splicing's roles in the context.

Splicing events often lead to enormous differences among isoforms in their sequences and structures and in the interactions, pathway networks, diseases, drugs, and organs formed. An enormous body of evidence has demonstrated the roles of alternative splicing in determining tissue-specific and species-specific differentiation patterns [2]. However, of interest is not only how it can respond to various signaling pathways, disease treatments and drug actions that target the splicing machinery but also what are the differences in pathways, diseases and drugs between different isoforms are generally overlooked. Therefore, it is crucial to the advance of basic and medical research that alternative splicing isoforms are interpreted and analyzed on a basis of context: pathway, disease, drug and organ because alternative splicing isoforms occur in a particular pathway, disease, drug action, or organ and we need to know about not only the isoforms themselves, but also their context regarding where they develop and stage.

We created the Synthetic Alternative Splicing Database (SASD) for users to detect specific alternative splicing isoforms and interpret their context at the pathway, disease, drug and organ level with maximum coverage and exclusive focus on alternative splicing. First, we extracted information on gene structures of all genes in the Ensembl Genes 71 database [19] and incorporated the IPAD database [20]. Then, we compiled artificial splicing transcripts. Lastly, we translated the artificial transcripts into alternative splicing peptides.

In addition, we built a web interface for users to browse 1) by genes/proteins, 2) by context (custom gene/protein set, signaling and metabolic pathway, disease, drug, and organ specificity).

In the end, we presented two case studies: 1) in liver cancer and 2) in breast cancer to demonstrate that the SASD can enable users to 1) identify novel alternative splicing isoform, and 2) analyze, characterize, and understand the impact of alternative splicing on genes involved in drug, disease, pathway, function, and organ-specificity.

The SASD, located at http://bioinfo.hsc.unt.edu/sasd is a comprehensive database containing 56,630 genes (Ensembl gene IDs), 95,260 transcripts (Ensembl transcript IDs), and 11,919,779 Alternative Splicing peptides (1,200,494 EXON_NM; 1,005,388 E_E_NM; 1,005,368 E_I_AS; 1,005,344 I_E_AS; 6,709,352 E_E_AS; and 993,833 INTRON_AS), and also covering about 1,956 pathways, 6,704 diseases, 5,615 drugs, and 52 organs incorporated from the IPAD [20].

It is the first comprehensive database that can be used for novel alternative splicing identification on the context of pathway, disease, drug and organ specificity or custom gene set. The maximum coverage and exclusive focus on alternative splicing provide enough sensitivity and specificity in identifying novel alternative splicing isoforms. The context specificity analysis enables us to improve our understanding of alternative splicing's roles in the context (custom gene set, pathway, disease, drug and organ specificity).

The SASD provides the scientific community with an efficient means to identify, analyze, and characterize novel Exon Skipping and Intron Retention protein isoforms from mass spectrometry data. We believe that it will be useful in annotating genome structures using rapidly accumulating proteomics data and will assist scientific research on signal transduction pathways regulating pre-mRNA, clinical therapy, disease prevention, and drug development.

## Results

### Database content statistics

The synthetic set of alternative splicing events (AS events) is derived from Ensembl gene annotation [19]. The Ensembl gene set includes both automatic and manual annotation, with all transcripts based on experimental evidence. Alternatively splice from transcripts of any given Ensembl gene are computationally synthesized and automatically annotated to provide a comprehensive list of six types of elementary alternative splicing events. These data can be searched on the website by gene, protein, transcript, peptide sequence, disease, organ, drug, and pathway. The AS events are available for the Homo sapiens. In order to reflect specific isoform in the context of pathway, disease, drug, and organ, the Integrated Pathway Analysis Database (IPAD) [20] is also incorporated. The IPAD [20] is the first comprehensive database for enrichment and inter-association analysis between pathway, disease, drug and organ. It was developed by integrating pathway, disease, drug, and organ specificity databases including BioCarta[21], KEGG[22], NCI-Nature curated[23], Reactome[24], CTD[25], PharmGKB[26], DrugBank[27], and Homer[28].

As of the current release (May 2013), SASD contains 56,630 genes (Ensembl gene IDs), 95,260 transcripts (Ensembl transcript IDs), and 11,919,779 Alternative Splicing peptides (1,200,494 EXON_NM; 1,005,388 E_E_NM; 1,005,368 E_I_AS; 1,005,344 I_E_AS; 6,709,352 E_E_AS; and 993,833 INTRON_AS) (Table 1), and also covers about 1,956 pathways, 6,704 diseases, 5,615 drugs, and 52 organs incorporated from the IPAD [20]. A comparison of alternative splicing in SASD against several common alternative splicing data sources is shown in Table 2.

**Table 1:** current statistics of database

Alternative Splicing Events	Count
EXON_NM	1,200,494
E_E_NM	1,005,388
E_I_AS	1,005,368
I_E_AS	1,005,344
E_E_AS	6,709,352
INTRON_AS	993,833
Total	11,919,779
Genes	56,630 (Ensembl gene ids)
Transcripts	95,260 (Ensembl transcript ids)

**Table 2:** a comparison of alternative splicing in SASD against several common alternative splicing data sources

	**ASTD**[14]	**EID**[15,16]	**ECgene**[18]	**PEPPI**[10]	SASD
AS coverage	9405	62,474	185,174	5,324,542	11,919,779
gene coverage	16,710	11,242	37,204	23,516	56,630
Last Updated	2008	2000	July 2007	Feb 2010	May 2013
Curation Type	Manual	Synthetic	Synthetic	Synthetic	Synthetic
Query by single gene	No	No	No	Yes	Yes
Query by Pathway	No	No	No	No	Yes
Query by Disease	No	No	No	No	Yes
Query by Drug	No	No	No	No	Yes
Query by Organ	No	No	No	No	Yes
Query by custom gene set	No	No	No	No	Yes
Query by gene sequence	No	No	No	Yes	Yes
Gene view	No	No	No	Yes	Yes
Transcript view	No	No	No	No	Yes
Region view	No	No	No	Yes	Yes
Peptide sequence	No	No	No	Yes	Yes

### General online features

**Figure 1:**
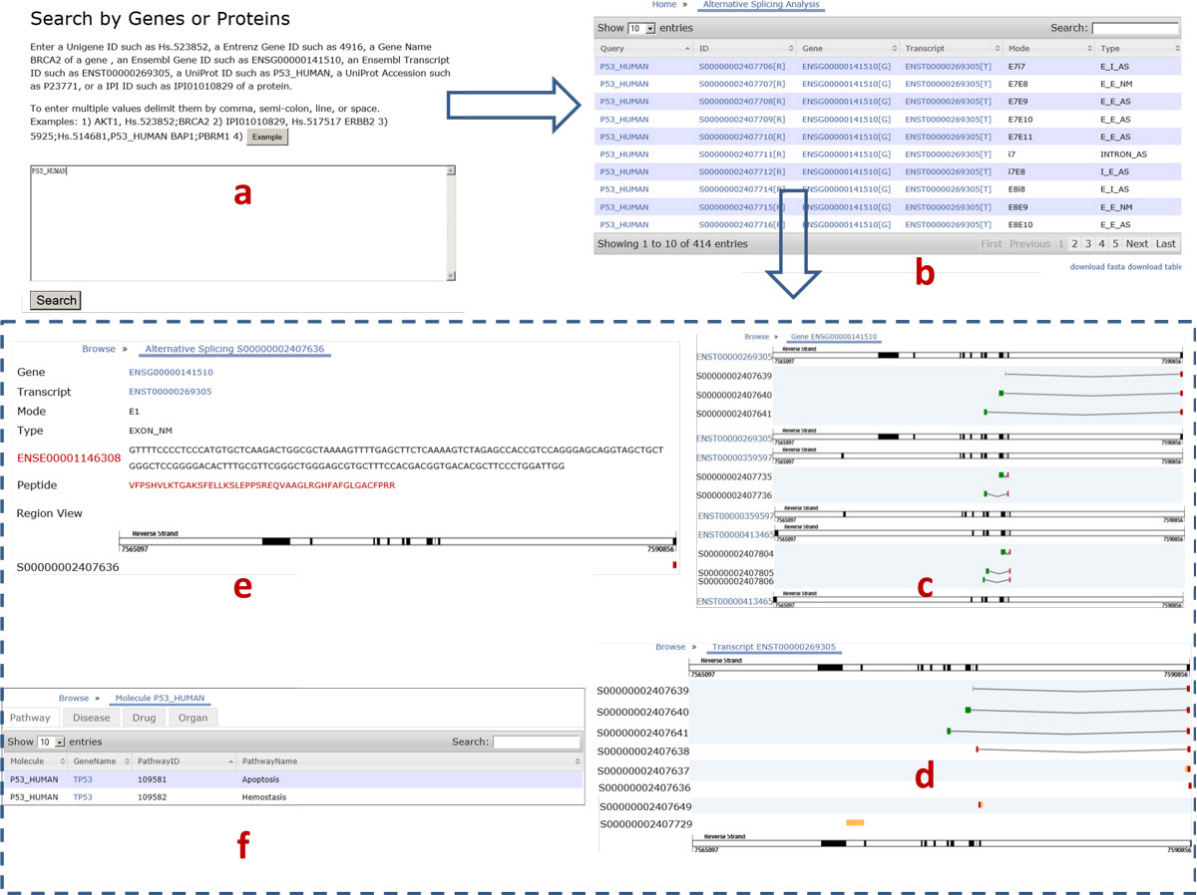
**Web interface structure**. a) Query by genes or proteins. For example, Ensembl Gene ID, Ensembl Transcript ID, UniGene IDs, Entrez gene IDs, gene names, UniProt IDs, UniProt Accessions or IPI IDs are all supported. To enter multiple values, delimit them by comma, semi-colon, line or space. b) search result. In the alternative splicing analysis table, it shows Query ID, SASD ID, Ensembl Gene ID, Ensembl Transcript ID, Mode, and Type. For each alternative splicing event, users can further browse its region view, gene view and transcript view by clicking on the links at the right corners of Column ID, Column Gene, and Column Transcript. c) gene view d) transcript view e) region view. f) molecule inter-association. It shows molecule, Gene Symbol, Pathway ID (Disease ID, Drug ID, Organ ID), and Pathway Name(Disease Name, Drug Name, Organ Name).

In Figure 1, we show the user interfaces of the web-based online version of SASD. It supports a powerful search options that allow users to specify a list of genes/proteins as the query input. Users can use different identifiers to query the database. The recommended terms are Ensembl gene ID, Ensemble transcript ID, Unigene ID's, Entrenz gene ID's, Gene name, Uniprot ID's, and IPI ID's. Users can enter a single gene or protein or multiple values delimited by comma, semi-colon, line, or space.

Some interesting features of SASD include the ability to be queried by multiple genes/proteins, pathway, disease, drug, and organ, to be searched by keyword in the Search Box over the table, and to support three different views for alternative splicing events: gene view, transcript view and region view.

**Figure 2:**
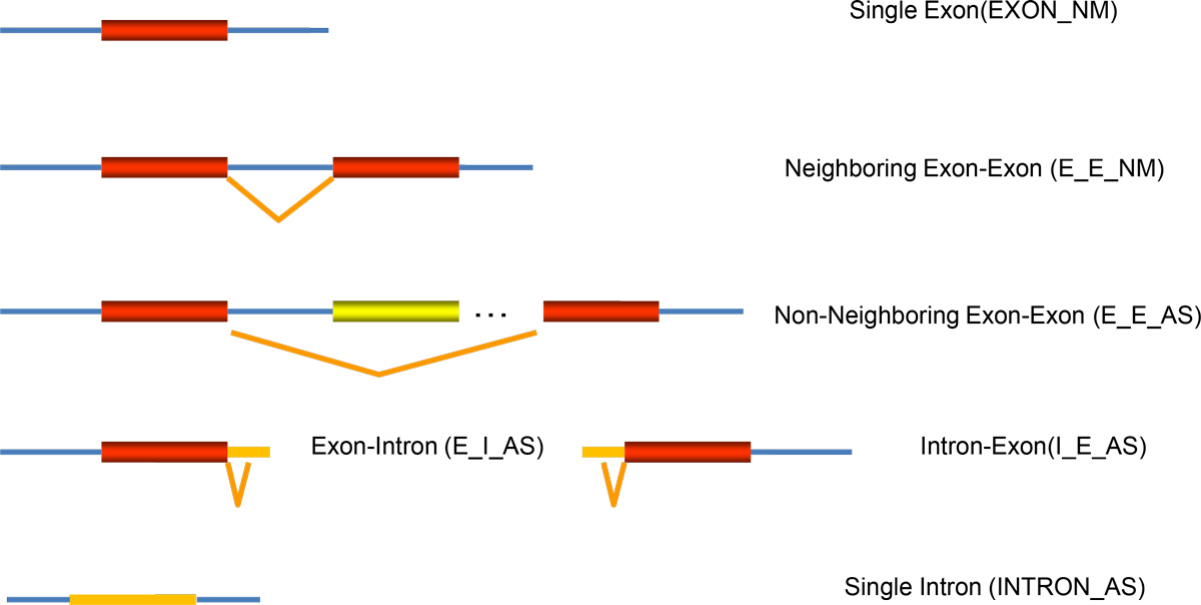
**Six types of as events**. The thick boxes represent exons and the thin boxes stand for introns. The orange lines stand for combination of exon-exon or exon-intron.

In response to the query input, SASD can retrieve a list of related alternative splicing events (gene, transcript, mode, type, and sequence) in a highly flexible table, with which users can further explore details about gene view, transcript view and region view of the alternative splicing events. For example, users can browse the gene view and transcript view by clicking on the link in the column of gene and transcript, respectively, and look through the genomic sequences of junction, splicing type, and peptide sequence in the region view by clicking on the R icon in the ID column. There are totally six types of alternative splicing events: EXON_NM, E_I_AS, E_E_NM, E_E_AS, INTRON_AS, and I_E_AS (Figure 2). AS stands for alternative splicing, and NM for normal splicing. Different colors are used to separate the junctions. Exon skipping event includes E_E_AS. Intron retention event includes E_I_AS, I_E_AS, and INTRON_AS. The Normal splicing is a normal RNA splicing, in which all introns are removed and the rest exons are joined into a contiguous sequence (http://www.dnalc.org/resources/animations/rna-splicing.html). In contrast, in the process of alternative splicing, particular exons or introns of a gene may be included within, or excluded from the final, processed messenger RNA produced from that gene (http://www.eurasnet.info/education/alternate-splicing/what-is-alternate-splicing). Noted: in E_I_AS, E_E_AS, INTRON_AS and I_E_AS, we use alternative splicing's original and narrow definition. But when we say SASD contains six types of alternative splicing, we use its generalized definition which includes not only narrowly defined alternative splicing but also normal splicing.

User queried alternative splicing data stored in SASD can also be downloaded as tab-delimited text and fasta format files when clicking on links below each alternative splicing table. The fasta format files can be run by any mass spectrometry search engine for alternative splicing identification.

### Case studies

We presented two case studies: 1)in liver cancer and 2) in breast cancer, to demonstrate that the SASD can enable users: 1) to identify novel alternative splicing isoform, and 2) to analyze, characterize, and understand the impact of alternative splicing on genes involved in drug, disease, pathway, function, and organ-specificity.

#### Case study 1: identification of novel alternative splicing isoforms

The Human Liver Proteome Project (HLPP) is a large-scale international collaborative initiative focusing on the proteomic analysis of the human liver. It aims to generate a comprehensive protein atlas of the human liver, uncover the proteomic basis of liver development, physiology and pathology and develop liver-specific diagnostics and therapeutics. We downloaded eight human fetal liver cytoplasm proteome data sets between weeks 16 and 24 of gestation available through the human liver proteome project web site (http://hlpic.hupo.org.cn). The human fetal liver between weeks 16 and 24 of gestation is a major site of fetal hematopoiesis and is at the critical turning point between immigration and emigration of the hematopoietic system. Protein profiling of its unique characteristics can help researchers understand the immigration and emigration process and improve conventional liver therapy [29].

**Figure 3:**
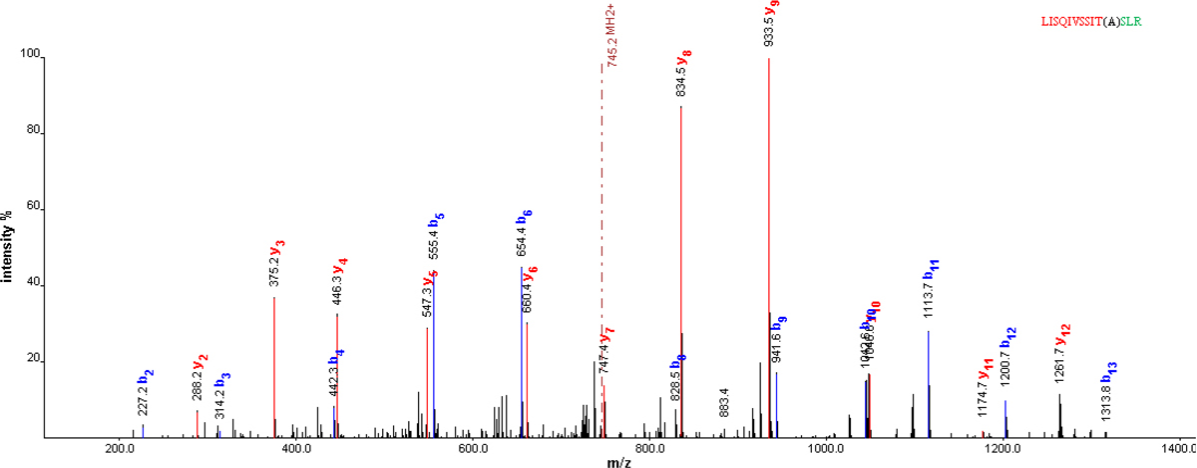
**Spectrum of LISQIVSSIT(A)SLR**. The blue lines represent b-ions. And the red lines represent y-ions.

OMSSA [30] is an efficient search engine for identifying MS/MS peptide spectra by searching libraries of known protein sequences. OMSSA scores significant hits with a probability score developed using classical hypothesis testing, the same statistical method used in BLAST. OMSSA searches protein libraries formatted for BLAST. In order to use OMSSA to identify alternative splicing isoforms, we first used the program formatdb to create our own alternative splicing sequence library from the fasta format file. Then we set OMSSA search parameters and run OMSSA to search the alternative splicing sequence library against the DTA files we merged from the eight raw liver proteome data. Totally, we identified 17 Novel alternative splicing isoforms which were undiscovered previously by the PEPPI [10] when we chose only peptides with at least two hits of samples as true peptides, of which 5 are left intron retention events, 2 right intron retention events, 6 single intron retention events, and 4 exon skipping events (Table 3). Bold text is the left part of the junction and italic text is the right part. Splicing site is marked by ^ or (). '()' means the splicing site is shared by the left region and right region. For example, the first peptide **LISQIVSSIT**(A)*SLR *is a synthetic product of the ENST00000473885 in gene ENSG00000243910 when its third intron is retained and combined together with its forth exon. The alanine is the shared splicing site between the intron and the exon. Although it can be mapped to ENSP00000449325, ENSP00000396212, ENSP00000412646, and ENSP00000443475, there is no hit when mapping it to ENSG00000243910's currently existed proteins. Thus it can be viewed as a novel alternative splicing isoform of ENSG00000243910. OMSSA search engine also provides a very good spectrum display and peak labeling and matching. For example, the matched MS/MS spectrum of the first peptide is shown in Figure 3.

**Table 3:** 17 novel peptide isoforms identified in human fetal liver project

hits	sequence	gene	transcript	mode	type
6	**LISQIVSSIT**(A)*SLR*	ENSG00000243910	ENST00000473885	i3E4	i_E_AS
3	**ELAEDGYSGVE**^*VR*	ENSG00000149273	ENST00000525690	E1i1	E_i_AS
2	**AIVAIENPADVSVISS**(R)	ENSG00000173163	ENST00000427417	i2E3	i_E_AS
2	**CLFKLSILIYSLGISV**(G)*QK*	ENSG00000069329	ENST00000299138	i2E3	i_E_AS
2	**DQEGQDVLLF**(I)*DNIFR*	ENSG00000110955	ENST00000547250	i2E3	i_E_AS
2	**DQEGQDVLLFID**^*NIFR*	ENSG00000110955	ENST00000552919	E6i6	E_i_AS
2	GAVLGAERPR	ENSG00000120251	ENST00000507898	i1	INTRON_AS
2	GTLYIIKLSADIR	ENSG00000115593	ENST00000419482	i8	INTRON_AS
2	**GVTIFVVL**(D)*ER*	ENSG00000170289	ENST00000320005	i11E12	i_E_AS
2	IGGIGTVPVGR	ENSG00000172244	ENST00000306862	i6	INTRON_AS
2	**INAVQISE**^*KIFR*	ENSG00000183091	ENST00000397345	E102E182	E_E_AS
2	**LL**^*AKTQNK*	ENSG00000119139	ENST00000377245	E15E21	E_E_AS
2	LPLQDVYK	ENSG00000172244	ENST00000306862	i6	INTRON_AS
2	SPGAWEGGREDR	ENSG00000160111	ENST00000291440	i2	INTRON_AS
2	**VSMILQSP**^*VLILR*	ENSG00000087274	ENST00000264758	E2E8	E_E_AS
2	**VTQWAEE**(R)	ENSG00000137177	ENST00000259711	E17E23	E_E_AS
2	WPDSQLAWFLR	ENSG00000119844	ENST00000238855	i8	INTRON_AS

Pathway enrichment analysis of the alternative splicing variants identified suggested that the involvement of these proteins especially in apoptosis. For example, the tight junction protein 2 (ENSG00000119139, TJP2) has been proposed to be a tumor suppressor gene. TJP2 protein and/or mRNA expression is either lost or decreased in pancreatic, prostate, breast and lung adenocarcinomas, in testicular in situ carcinoma and in lung squamous carcinoma. Exon 21 in the alternative splicing **LL**^*AKTQNK *(non-neighboring combination of exon 15 and exon 21) we identified has been involved in 3 of 5 isoforms previously identified.

#### Case study 2: identification of novel alternative splicing biomarkers

One of advantage of using SASD for alterative splicing analysis is that it supports query by pathway, disease, drug, organ, or user input gene set, which can be used for context specific alternative splicing analysis at the level of pathway, disease, drug, organ or any user specified gene set. In this case, we demonstrate how to use the new function of SASD to identify, analyze and characterize cancer-specific alternative splicing isoforms derived directly from cancer-specific genes. The 'specific' here means: 1) the genes from which the alternative splicing peptides are synthesized are linked to breast cancer in previous findings; and 2) the alternative splicing markers identified are significantly differentially expressed between breast cancer samples and normal samples.

Breast cancer is the fifth most common cause of cancer death (after lung cancer, stomach cancer, liver cancer and colon cancer). Among women worldwide, breast cancer is the most common cause of cancer death. In 2012, an estimated 226, 870 new cases of invasive breast cancer were expected to be diagnosed in women in the U.S., along with 63,300 new cases of non-invasive breast cancer. Traditional methods such as mammogram or biopsy can often detect a tumor only after a malignancy is advanced and may have metastasized to other organs. There is an urgent need for developing new methods for earlier detection of breast cancer. Cancer-specific splice isoforms has been observed in a few cases. Cancer-specific alterations in splice site selection can affect genes controlling cellular proliferation, cellular invasion, angiogenesis, apoptosis and multidrug resistance. Therefore, it is essential to develop cancer-specific alternative splicing isoform to enable biomarker discoveries for early detection of breast cancer.

We first downloaded 15 breast cancer related genes from the Cancer Gene Census[31]: AKT1, BAP1, BRCA2, CCND1, CDH1, EP300, ERBB2, ETV6, GATA3, MAP2K4, NTRK3, PBRM1, PIK3CA, RB1, and TP53. Then we created the 15 genes' alternative splicing sequence library from the fasta format file which we downloaded from the database SASD after a query by the 15 genes in the SASD. Lastly, we used the 15 breast cancer related genes' alternative splicing sequence library to run OMSSA search against 40 normal plasma and 40 breast cancer plasma. The plasma protein profiles of 40 samples from women diagnosed with breast cancer and 40 samples from healthy volunteer women as control were collected by the Hoosier Oncology Group (HOG) (Indianapolis, IN, USA). Most of patients involved were diagnosed with a stage II or III or earlier breast cancer.

The following options for OMSSA were used when identifying MS/MS peptide spectra: -e Trypsin (selecting trypsin to use for theoretical protein digestion), -y 1 (allowing maximum 1 missed cleavage), -hl 10 (**Maximum 10 peptide hitlist length per spectrum**), -he 0.1 (**EValue cutoff 0.1**), -x human (Homo sapiens to search), -te 2.0 (**Mass tolerance 2.0Da**), -tem monoisotopic (**Mass search type monoisotopic)**, -zl 1 (**Charge handle: low bound 1**), -zh 3 (**Charge handle: upper bound **3), -zt 3 (**Minimum charge to start using multiple charged products 3**), -to 0.8 (**Mass tolerance 0.8 Da**), -tom monoisotopic (mass search type monoisotopic), and -zoh 2 (**Maximum 2 charge state allowed for product ions**).

With the one-sided Wilcoxon signed-rank test [32], 8 alternative splicing markers (Table 4) were found differentially present (pvalue < 0.05) at cancer state, out of which there are five exon skipping, two single intron retention, and one left intron retention. All the 8 alternative splicing markers are not identified by the traditional alternative splicing database including the IPI database [12], the NCBI-nr database, and the UniProt knowledge base [33], and the PEPPI [10].

**Table 4:** 8 cancer-specific peptide markers identified in breast cancer

Peptide sequence	gene	transcript	mode	type	pvalue	h	c
SWGGRPQRMGAVPGGVWSAVLMGGAR	ERBB2	ENST00000269571	i18	INTRON_AS	9.48E-05	4	20
**QTPKHISESLGAEVDPDMSWSSSLATPPTLSSTVLI**(G)*LLHSSVK*	BRCA2	ENST00000380152	E7_E11	E_E_AS	8.57E-04	1	12
**SLWLQSQPHFCCFWLTVTFPPPLQ**^*THRELAQSSHAQR*	NTRK3	ENST00000317501	i2_E3	i_E_AS	1.22E-02	2	10
**WGLLLALLPPGAASTQ**(A)*VWTWMTR*	ERBB2	ENST00000269571	E1_E16	E_E_AS	1.22E-02	2	10
LSWNHVARALTLTQSLVSSVTSGK	NTRK3	ENST00000559764	i2	INTRON_AS	1.39E-02	4	13
**CQ**(G)*EPYHDIRFNLMAVVPDR*	BAP1	ENST00000460680	E3_E9	E_E_AS	3.33E-02	9	18
**QVLP**^*VGVLGPPGQQAPPPYPGPHPAGPPVIQQPTTPMFVAPPPK*	PBRM1	ENST00000296302	E9_E29	E_E_AS	3.89E-02	6	14
**DHLACW**^*DYDLCITCYNTKNHDHK*	EP300	ENST00000263253	E22_E31	E_E_AS	4.50E-02	4	11

Pathway analysis identified the cancer pathways including Pancreatic cancer, Pathways in cancer, Prostate cancer, Bladder cancer, Endometrial cancer, Non-small cell lung cancer, which are linked with the eight alternative splicing isoforms. The cancer-specific differentially expressed variants offer novel biomarker candidates that may function in breast cancer progression and metastasis. For example, the BRCA2 gene belongs to a class of genes known as tumor suppressor genes and is the most well-known gene linked to breast cancer risk. Bonnet et al. detected 20 variants of BRCA1 or BRCA2 that happened on exons 3, 16, 17, 18 or 25 from 17 index cases selected from families undergoing oncogenic consultations [34]. We identified a novel alternative splicing variant of BRCA2 **QTPKHISESLGAEVDPDMSWSSSLATPPTLSSTVLI**(G)*LLHSSVK *on the exon7 and exon11 as a non-neighboring exon-exon event.

This case study shows that compared to traditional alternative splicing database, the SASD can be more useful in identification of novel alternative splicing markers specific to some pathways, diseases, drugs or organ specification.

## Discussion

In this paper, we have demonstrated that SASD can be used to identify novel alternative splicing isoforms on the context of pathway, disease, drug and organ specificity or custom gene set. Its maximum coverage and exclusive focus on alternative splicing provide enough sensitivity and specificity in identifying novel alternative splicing isoforms. The context specificity analysis enables us to improve our understanding of alternative splicing's roles in the context (custom gene set, pathway, disease, drug and organ specificity). In Case Study 1, we illustrated the SASD's ability to identify novel alternative splicing isoform. In Case Study 2, we demonstrated how to use the new function of SASD to identify cancer-specific markers for distinguishing breast cancer from normal samples.

Alternative splicing is a widespread mechanism for generating protein diversity and regulating protein expression. Five basic types of alternative splicing events are generally recognized: 1) exon skipping, 2) intron retention, 3) mutually exclusive exons, 4) alternative donor site, and 5) alternative acceptor site. In exon skipping, an exon may be spliced out of the primary transcript or retained. This is the most common mode in mammalian pre-mRNAs. Intron retention is an event where a sequence is spliced out as an intron or remains in the mature mRNA transcript. Mutually exclusive exons event happens when one of two exons is retained in mRNAs after splicing, but not both. Alternative donor site is an event where an alternative 5' splice junction (donor site) is used, changing the 3' boundary of the upstream exon. And alternative acceptor site is an event where an alternative 3' splice junction (acceptor site) is used, changing the 5' boundary of the downstream exon.

The SASD does not contain the last three types of events. But actually, they all can be derived indirectly from the two basic types: exon skipping and intron retention which are included in the SASD in the form of E_E_AS, and E_I_AS, INTRON_AS, and I_E_AS, respectively. For example, if two modes such as E1E3 and E2E4 happen at the same time, it is actually a mutually exclusive exons event. Alternative 5' donor site can be detected by the type E_I_AS such as E1i1 in the SASD where donor site i1 is remained. Alternative 3' acceptor site can be detected by the type I_E_AS such as i4E5 in the SASD where acceptor site i4 is remained.

In addition, the SASD contains EXON_NM, E_E_NM, and INTRON_AS. The two normal splicing types: EXON_NM and E_E_NM are included as a contrast to the alternative splicing events. The INTRON_AS is actually a complement of intron retention.

Some artificially synthetized peptides in the database may not exist biologically. This will reduce computational efficiency but won't affect the usage of the database, as long as the following assumptions are met: if some artificially synthetized peptides in the database don't actually exist biologically, they will less likely or never match with proteome experimental spectra.

The SASD is similar to a modified peptide database for Post Translational Modification (PTM) identification from MS/MS. The first approach to PTM identification proposed by Yates et al. [35], enumerating all possible modifications for each peptide from the database still works very well with small database. Enumerating all possible mutations and modifications in the database makes the database prohibitively large and is computationally expensive, so that using this kind of database to search for modifications remains limited to smaller databases.

One advantage of using SASD is context-based alternative splicing identification. Users can build a relatively small database based on the context (pathway, disease, disease, drug, and other user input gene set). This feature enables users not only to identify context-specific alternative splicing, but also with significantly-improved computational efficiency.

Fortunately, all search engines provide their own scores as thresholds such as expectation value which is the number of matches with equal or better scores that are expected to occur by chance alone. In order to increase the true discovery rate for these artificially synthetized peptides that don't exit biologically and that is less likely or never to be identified by proteome experiments, when using SASD, we recommend to use 1) the p-value (Mascot) or evalue (OMSSA) as thresholds provided by various search engines and 2) cross-validation experiments. That is, if a synthetic AS peptide is identified with significant score and from more than n samples (for example, *n *= 2), we say the synthetic AS peptide is identified and the synthetic AS peptide exists biologically. More stringent threshold or more experiment validations from other labs or by other methods such as PCR are definitely needed for further validation because SASD is an in-silico database after all.

## Methods

### Database pipeline

The overall pipeline of SASD comprises three steps: 1)extracting information on gene structures of all genes in the human genome and incorporating the IPAD database [20], 2)compiling artificial splicing transcripts, and 3) translating the artificial transcripts into alternative splicing peptides.

In the first step, we use the BioMart to extract information on all human genes in the Ensembl [19] from the Homo sapiens genes dataset (GRCh37.p10) in the Ensembl Genes 71 database. We then extract information on each human gene's position, name, exon/intron coordinates, exon phase, sequences, and annotation. The information is organized in a relational database hosted in a local SQL server 2012 database server.

In the second step, we generate artificial splicing transcript (AST), which is an exhaustive compilation of two categories of peptides (the first is the peptides translated from all single exons and introns, the second is the peptides that covers all theoretically possible exon/intron junction regions of all genes in the human genome). With these two categories of peptides, both the whole sequence of the genome and all possibilities of alternative splicing are covered. In addition to single exon(EXON_NM) and single intron (INTRON_AS), four types of exon/intron sequence junctions are considered when generating ASTs: intron-exon (I_E_AS, left intron retention junction), exon-intron (E_I_AS, right intron retention junction), neighboring exon-exon (E_E_NM, normal splicing junction) and non-neighboring exon-exon (E_E_AS, exon skipping junction). For each type, 120 nucleotides both upstream and downstream of the joined sequence beside the junction site are extracted, resulting in a computationally synthesized virtual transcript of 240 nucleotides long. Determination of the number 120 is based on the length distribution of fragment obtained from protein digestion in MS/MS experiments. The boundaries where two components are spliced are known as splicing sites. Totally, there are three types of common splicing events: Normal Splicing, Exon Skipping, and Intron Retention in the SASD. The Normal Splicing includes single exon (EXON_NM) and neighboring exon-exon junction (E_E_NM), the Exon Skipping includes non-neighboring exon-exon junction (E_E_AS), and the Intron Retention includes single intron (INTRON_AS), left intron retention junction (I_E_AS), right intron retention junction (E_I_AS).

In the third step, we directly use the phase to translate the sequence for the exons with the phase information in Ensembl transcript. For the exons without the phase information in Ensembl transcript, three translations are first derived, each of which corresponds to a possible opening reading frame (ORF) and generates one peptide. Then, the automatic translation procedure calculates the length of the peptide that map across the splicing site. Lastly, the translation which contains the longest peptide is reserved as alternative splicing peptide for SASD.

### Online SASD server design

The online version of SASD database is a typical 3-tier web application [10], with an SQL Server2012 database at the backend database service layer as Data Access Tier, Apache/PHP server scripts to the middleware application web server layer as Logic or Application Tier, and CSS-driven web pages presented on the browser as Presentation Tier.

**Figure 4:**
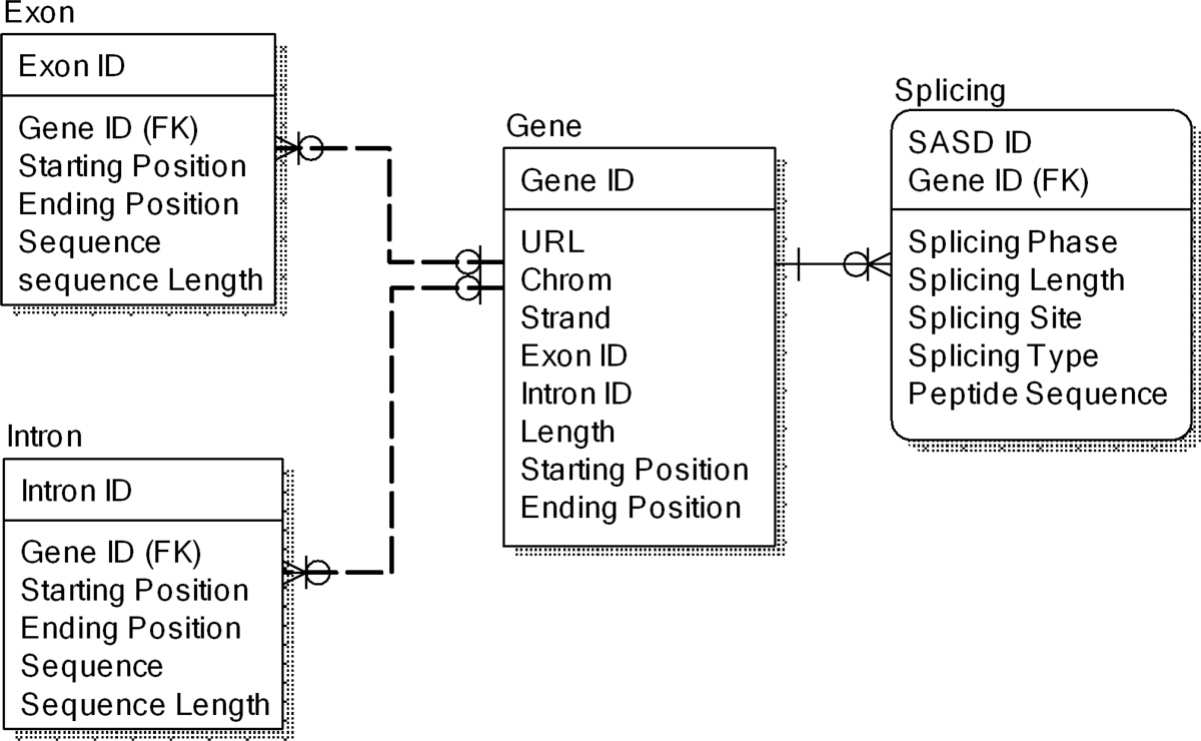
**Relational metadata model**. The datasets were derived by the data generation pipeline.

The result tables derived from the data generation steps are imported into the SQL Server2012 database (Figure 4). The pathway-gene, disease-gene, drug-gene, organ-gene, protein-gene, gene-gene mapping tables enable users to query the database with different IDs.

### Pathway analysis

Pathway analysis is performed using the following databases: Integrated Pathway Analysis Database (IPAD) (http://bioinfo.hsc.unt.edu/ipad/) [36].

## Conclusion

We developed SASD as a complement to the currently existing alternative splicing databases to perform novel alternative splicing identification on a biological context such as pathway, disease, drug and organ specificity or custom gene set with maximum coverage and exclusive focus on alternative splicing. SASD integrates the gene structure from Ensembl [19] and the context (pathway, disease, drug and organ specificity) from IPAD [20].

A single gene/transcript/protein, custom gene set, pathway, disease, drug, organ related alternative splicing can be searched, displayed, and downloaded from our online user interface. The current SASD database can help users discover novel alternative splicing from mass spectrometry and interpret them at the context of pathway, disease, drug and organ specificity or custom gene set with maximum coverage and exclusive focus on alternative splicing. We believe that it could help generate novel hypothesis on molecular risk factors and molecular mechanisms of complex diseases, leading to identification of potentially highly specific protein biomarkers. Lastly, our database was demonstrated by comparison to other known databases and two case studies.

## Competing interests

The authors declare that they have no competing financial interests.

## Authors' contributions

RD conceived the initial work and designed the method for the database construction. FZ generated the datasets, developed the statistics method, the database backend and the web-based interface, and performed the alternative splicing analyses for the case studies. All authors are involved in the drafting and revisions of the manuscript.

## Declarations

The publication costs for this article were funded by the bioinformatics program in University of North Texas Health Science Center.

This article has been published as part of *BMC Bioinformatics *Volume 14 Supplement 14, 2013: Proceedings of the Tenth Annual MCBIOS Conference. Discovery in a sea of data. The full contents of the supplement are available online at http://www.biomedcentral.com/bmcbioinformatics/supplements/14/S14.
